# CNA WORK IS BETTER LEARNED HANDS-ON: TRAINING CERTIFIED NURSING ASSISTANTS TO FEED RESIDENTS WITH DEMENTIA

**DOI:** 10.1093/geroni/igz038.2456

**Published:** 2019-11-08

**Authors:** Joy W Douglas, Seung Eun Jung, Hyunjin Noh, Amy Ellis, Christine Ferguson

**Affiliations:** 1 University of Alabama, Tuscaloosa, Alabama, United States; 2 The University of Alabama School of Social Work, Tuscaloosa, Alabama, United States

## Abstract

In nursing homes across the United States, Certified Nursing Assistants (CNAs) provide essential mealtime assistance to residents with dementia who have difficulty feeding themselves. However, dementia-related training content in CNA programs can vary. In this qualitative study, we sought to understand the training provided to CNAs in Alabama, and to identify the ideal training modality for content related to feeding residents with dementia. Nine focus groups were conducted with 53 CNAs. Each participant had at least one year of working experience as a CNA caring for older adults. Focus groups were audio recorded and transcribed verbatim. Data were analyzed using the directed content analysis approach. Analyses revealed several key themes related to training CNAs to feed residents with dementia. Across focus groups, CNAs agreed that they needed additional training about feeding residents with dementia. They unanimously agreed that the best person to provide such training should be an experienced CNA, not a nurse or other healthcare provider. In terms of delivery, they preferred hands-on training and role playing. CNAs also emphasized that while some learning takes place in a didactic setting, the most valuable learning moments involve on-the-job experience in feeding residents, where they are mentored by seasoned CNAs. Findings from this study revealed the need for providing CNAs training on feeding residents with dementia. CNA training programs that includes hands-on activities mentored by seasoned CNAs could increase CNAs’ ability to provide optimal meal assistance to nursing home residents with dementia.

